# Supplementary material for: Short reads from honey bee (Apis sp.) sequencing projects reflect microbial associate diversity
Source: PeerJ. 2017 Jul 12;5:e3529. doi: 10.7717/peerj.3529 (PMC5510586; doi:10.7717/peerj.3529)
Supplement: Figure S1 — All short reads from this dataset were mapped against Lactobacillus 16S reference sequences as detailed in the ‘Materials & Methods’ section. Thus retrieved 16S sequences are highlighted with thick, dark blue lines. All other taxa in this tree are identical to the ones in Fig. 2A, as is the color scheme. Although the topology differs between these two Lactobacillus trees, it is evident that the strains recovered from the Engel, Martinson & Moran (2012) dataset cluster within the Firm-4 and Firm-5 Lactobacillus groups. Engel, Martinson & Moran (2012) essentially find the same (“These distinct clusters reflect the eight dominant species with the two closely related Firmicutes (Firm-4 and Firm-5) [...]”; see also their Fig. 1C) using the programs MetaPhyler ( http://metaphyler.cbcb.umd.edu/) and IMG/M ( https://img.jgi.doe.gov/) for taxonomic profiling. [file peerj-05-3529-s001.pdf]

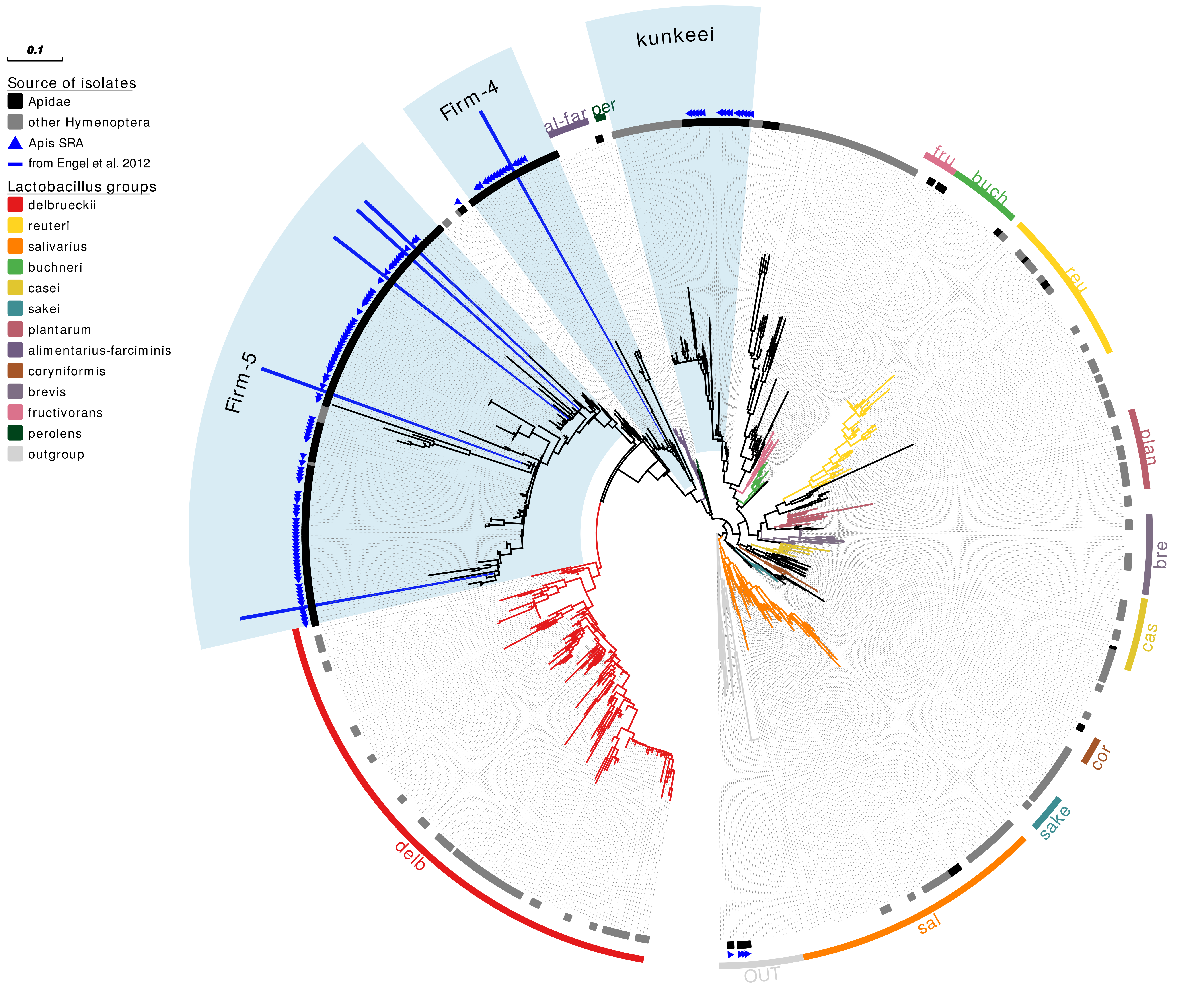

0.1
Source of isolates
Apidae
other Hymenoptera
Apis SRA
from Engel et al. 2012
Lactobacillus groups
delbrueckii
reuteri
salivarius
buchneri
casei
sakei
plantarum
alimentarius-farciminis
coryniformis
brevis
fructivorans
perolens
outgroup
kunkeei
Firm-4
Firm-5
al-far
per
fru
buch
reu
plan
bre
cas
cor
sake
sal
OUT
delb
