# Supplementary figures and images for: Short reads from honey bee (Apis sp.) sequencing projects reflect microbial associate diversity
Source: PeerJ. 2017 Jul 12;5:e3529. doi: 10.7717/peerj.3529 (PMC5510586; doi:10.7717/peerj.3529)

log2(RPKM)

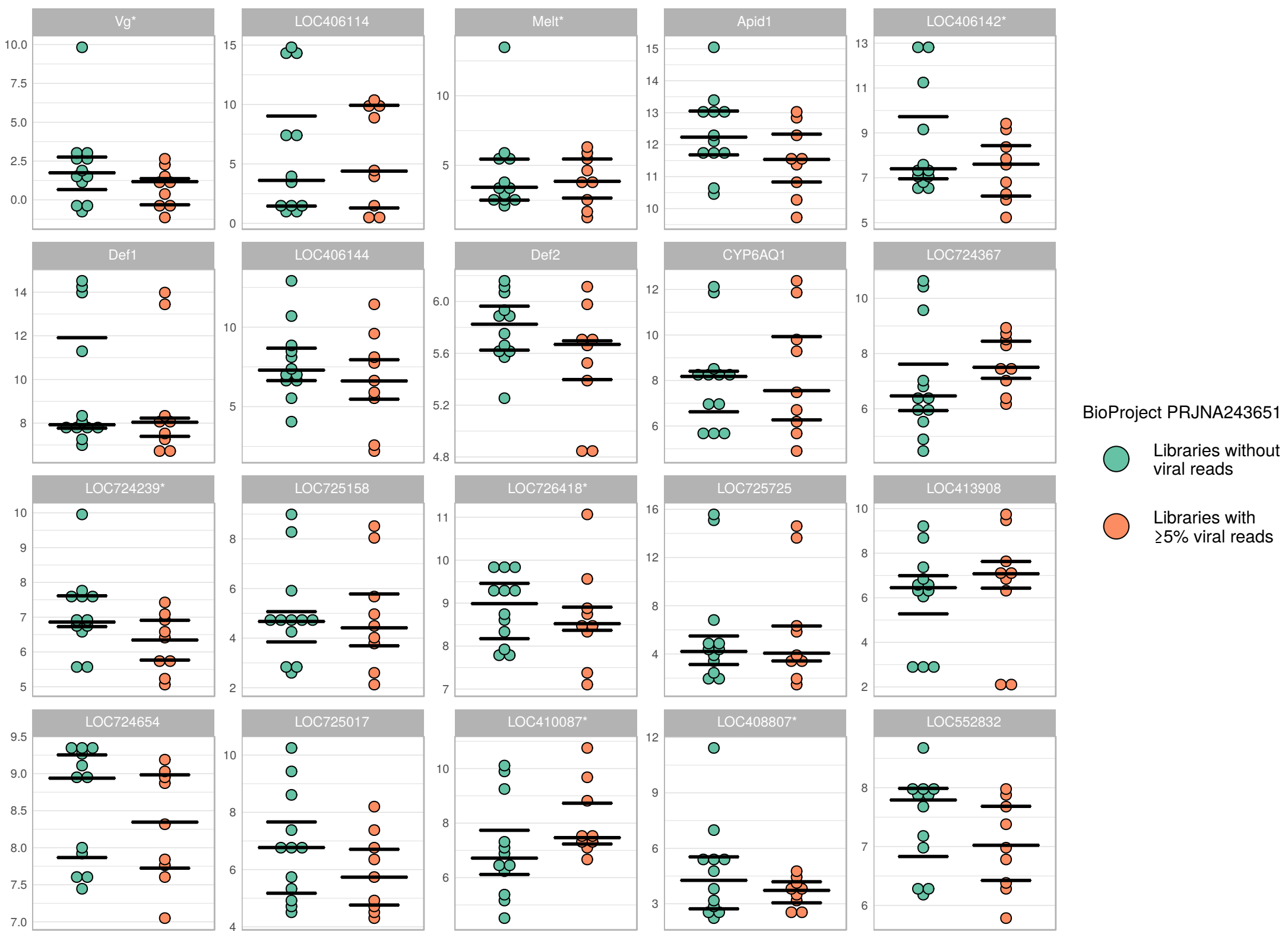

log2(RPKM)

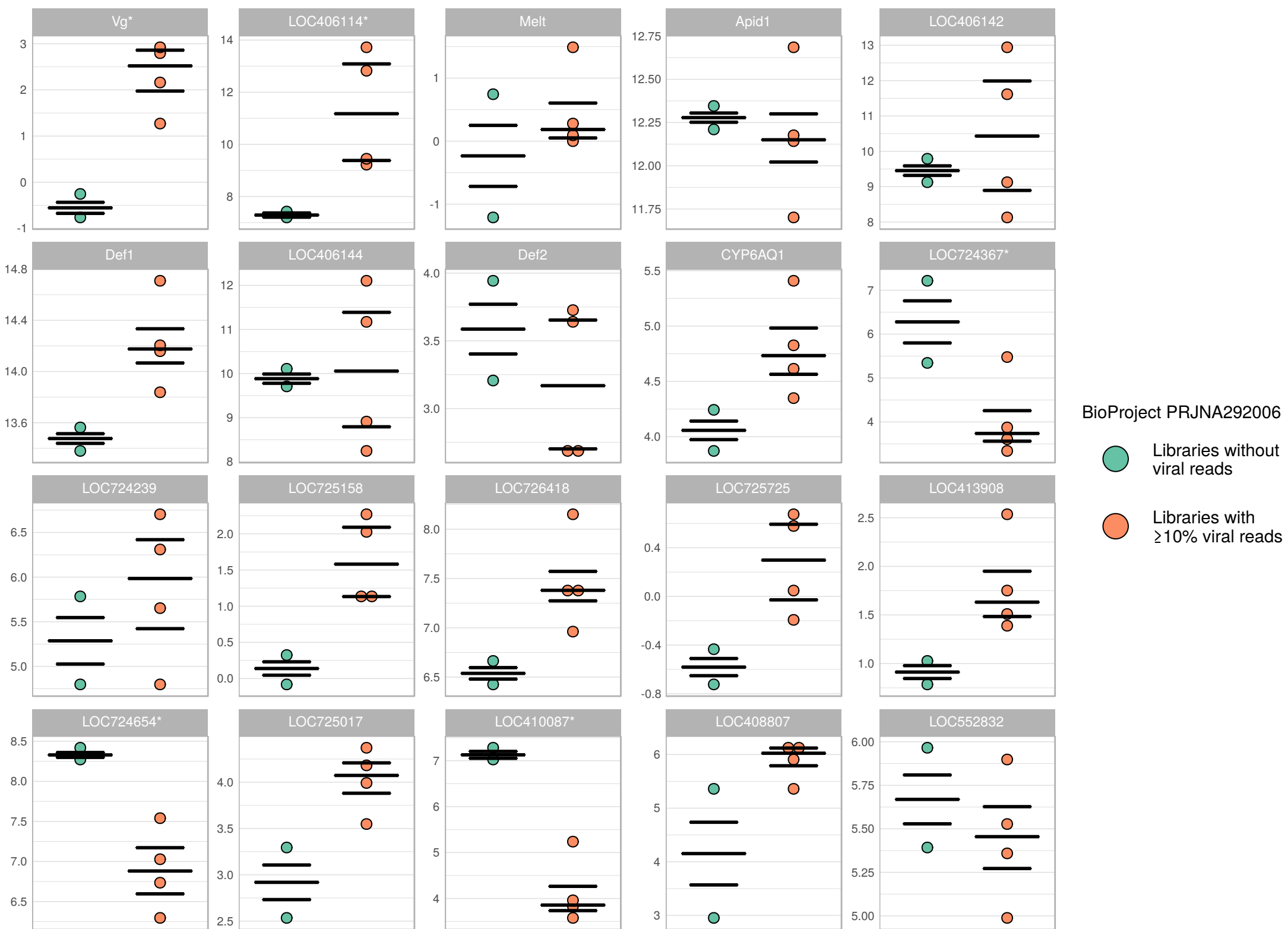

log2(RPKM)

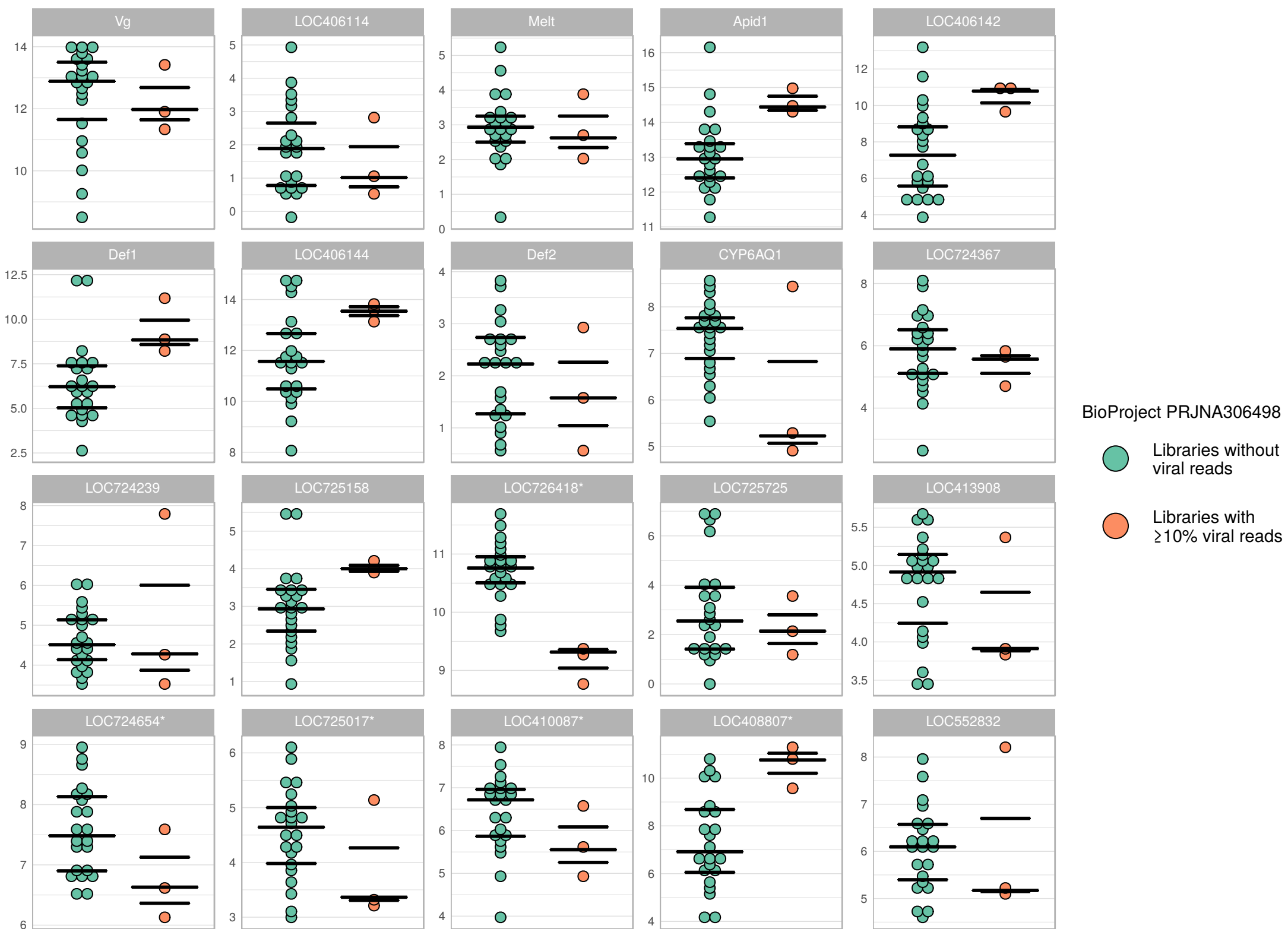

log2(RPKM)

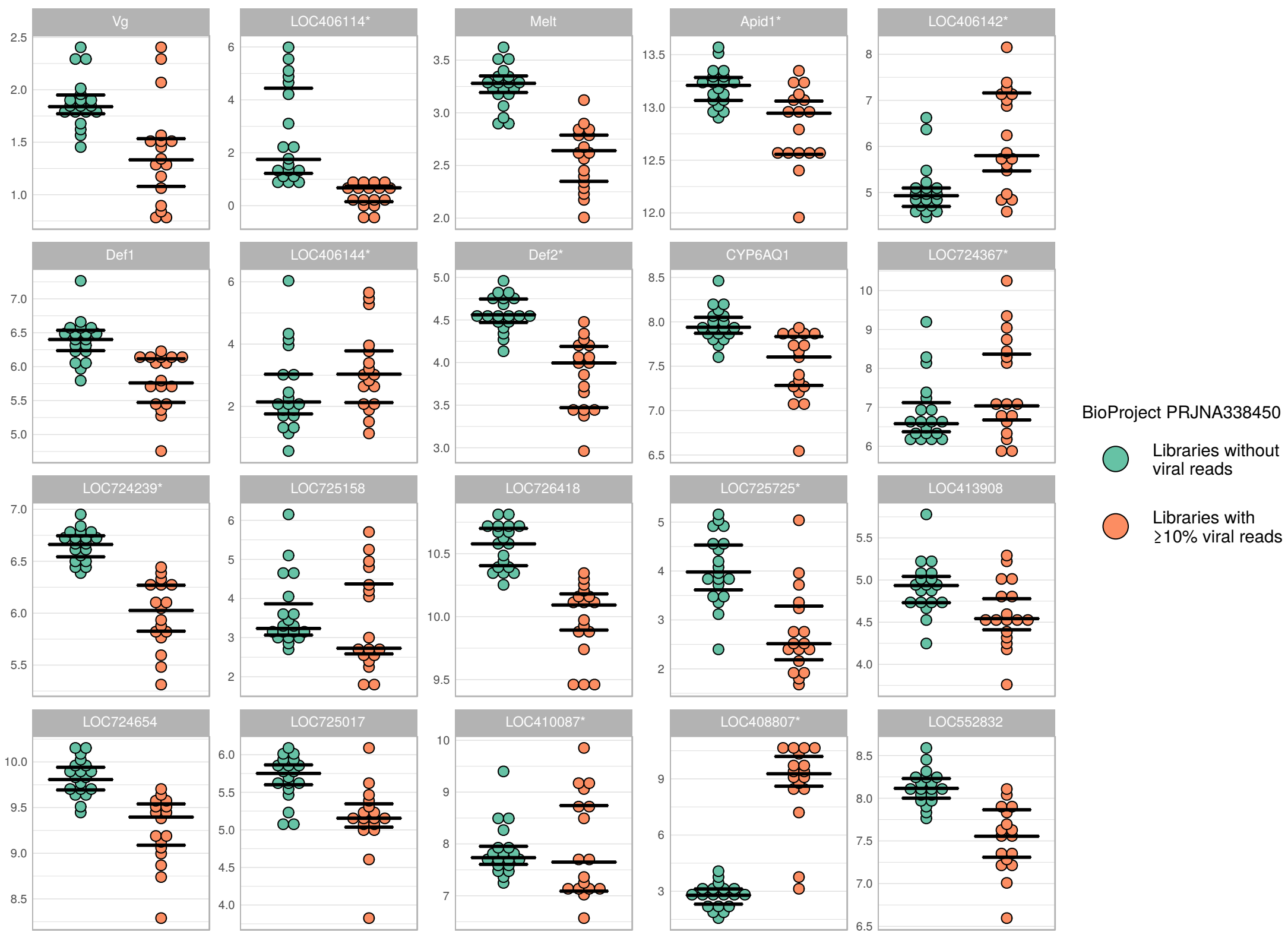

Supplement: Supplemental Information 1 — Differentially expressed genes as determined with edgeR are marked with an asterisk. See ‘Materials & Methods’ for more details. [file peerj-05-3529-s002.pdf]
