# Supplementary material for: Short reads from honey bee (Apis sp.) sequencing projects reflect microbial associate diversity
Source: PeerJ. 2017 Jul 12;5:e3529. doi: 10.7717/peerj.3529 (PMC5510586; doi:10.7717/peerj.3529)
Supplement: Table S3 [file peerj-05-3529-s005.pdf]

Name of strain as used in MS	NCBI BioProject accession number
Fructobacillus ficulneus	PRJDB3977
Fructobacillus fructosus	PRJDB3977
Fructobacillus pseudoficulneus	PRJDB3977
Fructobacillus sp. EFB-N1	PRJNA284658
Fructobacillus tropaeoli	PRJDB3977
Lactobacillus apinorum Fhon13	PRJNA270968
Lactobacillus delbrueckii	PRJNA16871
Lactobacillus kunkeei Fhon2	PRJNA270967
Lactobacillus kunkeei LAan	PRJNA270961
Lactobacillus kunkeei LAce	PRJNA270962
Lactobacillus kunkeei LAdo	PRJNA270963
Lactobacillus kunkeei LAfl	PRJNA270964
Lactobacillus kunkeei LAko	PRJNA270965
Lactobacillus kunkeei LAla	PRJNA270966
Lactobacillus kunkeei LAni	PRJNA270969
Lactobacillus kunkeei LAnu	PRJNA270970
Lactobacillus kunkeei LMbe	PRJNA270972
Lactobacillus kunkeei LMbo	PRJNA270973
Lactobacillus kunkeei YH-15	PRJNA270974
Leuconostoc gelidum	PRJNA174297
Leuconostoc mesenteroides	PRJNA315
Spiroplasma apis B31	PRJNA184751
Spiroplasma atrichopogonis GNAT3597	PRJNA253349
Spiroplasma cantharicola CC-1	PRJNA253642
Spiroplasma chrysopicola DF-1	PRJNA184747
Spiroplasma culicicola AES-1	PRJNA184749
Spiroplasma diminutum CUAS-1	PRJNA184745
Spiroplasma eriocheiris DSM 21848	PRJNA253647
Spiroplasma kunkelii CR2-3x	PRJNA270865
Spiroplasma litorale TN-1	PRJNA253651
Spiroplasma melliferum IPMB4A	PRJNA80357
Spiroplasma melliferum KC3	PRJNA32299
Spiroplasma mirum ATCC 29335	PRJNA217816
Spiroplasma poulsonii MSRO	PRJNA256019
Spiroplasma sabaudiense Ar-1343	PRJNA184750
Spiroplasma syrphidicola EA-1	PRJNA184748
Spiroplasma taiwanense CT-1	PRJNA184744
Spiroplasma turonicum Tab4c	PRJNA291635
