# Supplementary material for: Short reads from honey bee (Apis sp.) sequencing projects reflect microbial associate diversity
Source: PeerJ. 2017 Jul 12;5:e3529. doi: 10.7717/peerj.3529 (PMC5510586; doi:10.7717/peerj.3529)
Supplement: Table S6 [file peerj-05-3529-s008.pdf]

Locus	Description	PRJNA243651				PRJNA292006				PRJNA306498				PRJNA338450			
		logFC	logCPM	PValue	FDR	logFC	logCPM	PValue	FDR	logFC	logCPM	PValue	FDR	logFC	logCPM	PValue	FDR
Apid1	apidaecin 1	-0.513	17.377	0.276	0.501	-0.580	17.420	0.482	0.741	0.846	17.223	0.074	0.211	0.139	18.941	0.004	0.009
CYP6AQ1	cytochrome P450 6AQ1	0.832	16.217	0.329	0.548	0.280	11.025	0.701	0.876	-1.147	12.518	0.060	0.201	0.041	14.999	0.402	0.447
Def1	defensin 1	-0.346	17.948	0.818	0.909	0.196	18.995	0.861	0.957	1.281	12.323	0.172	0.246	-0.123	11.979	0.162	0.231
Def2	defensin 2	0.085	10.678	0.808	0.909	-1.036	8.255	0.114	0.321	-0.871	5.730	0.146	0.244	-0.210	9.890	0.003	0.009
LOC406114	alpha-amylase	-3.435	18.801	0.067	0.168	5.377	18.605	0.000	0.000	-1.246	7.637	0.289	0.340	-2.821	9.922	0.000	0.000
LOC406142	hymenoptaecin	-2.326	15.080	0.017	0.083	1.354	15.820	0.144	0.321	1.123	13.009	0.209	0.279	1.836	11.912	0.000	0.000
LOC406144	abaecin	-0.539	13.201	0.578	0.757	0.389	14.667	0.666	0.876	0.858	15.257	0.167	0.246	1.058	8.616	0.021	0.041
LOC408807	uncharacterized LOC408807	-3.198	12.453	0.002	0.018	1.051	11.578	0.137	0.321	2.167	13.299	0.003	0.062	7.133	15.399	0.000	0.000
LOC724367	protein lethal(2)essential for life-like	0.382	13.655	0.543	0.757	-2.751	11.518	0.000	0.000	-1.280	10.400	0.128	0.244	1.383	14.121	0.000	0.001
LOC410087	uncharacterized LOC410087	1.277	14.768	0.032	0.129	-3.255	12.853	0.000	0.000	-1.351	11.713	0.045	0.181	0.785	15.411	0.001	0.005
LOC413908	cytochrome P450 6A1	0.854	13.619	0.219	0.438	0.626	7.993	0.379	0.688	-0.860	9.793	0.089	0.222	0.223	11.959	0.089	0.148
LOC552832	glycine N-methyltransferase	0.019	13.360	0.934	0.983	-0.729	11.701	0.257	0.515	0.060	10.911	0.835	0.835	-0.087	14.722	0.104	0.160
LOC724239	kynurenine/alpha-aminoadipate aminotransferase, mitochondrial-like	-0.967	13.914	0.049	0.139	0.056	12.456	0.962	0.962	0.739	10.332	0.127	0.244	-0.153	13.881	0.002	0.008
LOC724654	cytochrome b5 type B-like	0.138	13.423	0.585	0.757	-1.994	12.754	0.005	0.019	-1.319	11.213	0.012	0.083	-0.065	15.303	0.203	0.271
LOC725017	uncharacterized LOC725017	-0.927	14.416	0.125	0.278	0.571	11.028	0.418	0.697	-1.038	10.602	0.037	0.181	-0.022	13.634	0.824	0.824
LOC725158	peptidoglycan recognition protein S1	0.461	12.244	0.606	0.757	1.153	6.704	0.117	0.321	0.424	7.340	0.334	0.372	0.398	10.101	0.235	0.283
LOC725725	uncharacterized LOC725725	-0.082	17.895	0.994	0.994	0.246	4.916	0.766	0.901	-2.585	8.299	0.138	0.244	-0.657	9.449	0.019	0.041
LOC726418	flavin-containing monooxygenase FMO GS-OX-like 3-like	1.051	16.169	0.046	0.139	0.467	14.041	0.584	0.835	-2.492	16.642	0.008	0.083	-0.065	17.953	0.240	0.283
Melt	melittin	-4.287	12.761	0.003	0.022	-0.094	4.700	0.953	0.962	-1.137	6.349	0.260	0.325	-0.165	7.956	0.052	0.095
Vg	vitellogenin	-4.210	12.831	0.000	0.010	2.795	10.015	0.000	0.001	-0.919	19.756	0.402	0.423	0.078	10.557	0.574	0.604
